# Integrated Effects of Two Additives on the Enhanced Performance of PTB7:PC_71_BM Polymer Solar Cells

**DOI:** 10.3390/ma9030171

**Published:** 2016-03-05

**Authors:** Lin Wang, Suling Zhao, Zheng Xu, Jiao Zhao, Di Huang, Ling Zhao

**Affiliations:** 1 Key Laboratory of Luminescence and Optical Information, Beijing Jiaotong University, Ministry of Education, Beijing 100044, China; 13121569@bjtu.edu.cn (L.W.); zhengxu@bjtu.edu.cn (Z.X.); 14118436@bjtu.edu.cn (J.Z.); 2008-hd@163.com (D.H.); 13118413@bjtu.edu.cn (L.Z.); 2 Institute of Optoelectronic Technology, Beijing Jiaotong University, Beijing 100044, China

**Keywords:** organic solar cells, mixed additives, absorption, hole mobility

## Abstract

Organic photovoltaics (OPVs) are fabricated with blended active layers of poly [[4,8-bis[(2-ethylhexyl)oxy]benzo[1,2-b:4,5-b']dithiophene-2,6-diyl][3-fluoro-2-[(2-ethylhexyl)carbonyl] thieno[3,4-b]thiophenediyl]]: [6,6]-phenylC71-butyric acid methyl ester (PTB7:PC_71_BM). The active layers are prepared in chlorobenzene (CB) added different additives of 1, 8-Diiodooctane (DIO) and polystyrene (PS) with different concentrations by spin coating. A small addition, 0.5%–5% by weight relative to the BHJ components, of inert high molecular weight PS is used to increase the solution viscosity and film thickness without sacrificing desirable phase separation and structural order. The effects of the PS are studied with respect of photovoltaic parameters such as fill factor, short circuit current density, and power conversion efficiency. Together with DIO, the device with 3.0 v% DIO and 1 wt % PS shows a high power conversion efficiency (PCE) of 8.92% along with an open-circuit voltage (*V_oc_*) of 0.76 V, a short-circuit current (*J_sc_*) of 16.37 mA/cm^2^, and a fill factor (FF) of 71.68%. The absorption and surface morphology of the active layers are investigated by UV-visible spectroscopy, atomic force microscopy (AFM) respectively. The positive effect of DIO and PS additives on the performance of the OPVs is attributed to the increased absorption and the charge carrier transport and collection.

## 1. Introduction

Molecular species with well-defined structures [1,2,3] are being considered as possible substitutions for conjugated polymer counterparts in the fabrication of bulk heterojunction (BHJ) organic photovoltaics (OPVs) [4,5,6]. High power conversion efficiencies (PCEs) have been achieved in solution-processed molecular solar cells through a combination of chemical design and deposition methods with optimized morphology [7]. Despite the advantages of the structural precision [8] and purity of materials [9,10,11], some challenges to control the thickness and morphology [12,13] of the active layer decided by the processing conditions are critical to the properties of solar cells, which reasonably influences the light absorption and recombination of carriers [14]. A representative example involves the blends comprised of PTB7/PC_71_BM, which is one of the highest-performing systems, based on the addition of small quantities of a high boiling point additive such as diiodooctane (DIO), a kind of commonly used additive [15] to meliorate the morphology of the blend film [16] and promote the phase separation [17]. However, the functions of DIO are still a subject of debate in both polymer and small molecule systems [18], but in the case of PTB7/PC_71_BM it is known to improve the charge transporting by increasing the final crystalline content of the film and allowing the donor phase more than one polymorph during the film formation [19,20]. Nonetheless, adding DIO into the blend film cannot improve the light absorption of the blend film. Even worse, up to now, there is still little research about the negative effect of DIO additives on performance of OPVs [21].

Another effective strategy for PCEs improvement of OPVs is adding a high molecular polystyrene (PS) into the pristine active layer. PS can increase not only increase the pristine solution viscosity but also the film thickness without sacrificing desirable phase separation [7] and structural order and decrease the recombination of electron-hole pairs in the blend film [22,23]. At the same time, PS can improve the light absorption of the blend film. Therefore, in this contribution, polystyrene (PS) was used to fabricate the BHJ polymer solar cell based on PTB7/PC_71_BM as the active layer. DIO and/or PS were varied with different ratios during the solution preparation of the organic active layer. The effect of the PS was investigated in PTB7:PC_71_BM blended films. The morphology of the active layer with different additive ratios has been studied and the related OPVs device performance also has been reported.

## 2. Experimental Section

### 2.1. Fabrication of Solar Cells

Devices used materials that were used as purchased. Poly (3,4-ethylenedioxythiophene): poly (styrenesulfonate) (PEDOT:PSS), poly[[4,8-bis[(2-ethylhexyl)oxy]benzo[1,2-b:4,5-b'] dithiophene-2,6-diyl][3-fluoro-2-[(2-ethylhexyl) carbonyl]thieno[3,4-b]thiophenediyl]](PTB7) with a molecular weight of ~200 kg/mol and polydispersity of ~4, [6,6]-phenylC71-butyric acid methyl ester(PC_71_BM), and polystyrene(PS) with a molecular weight of ~370 kg/mol were purchased from Clevios P, 1-Material INC, Nano-C company and Sigma-Aldrich Corporation respectively. PTB7 and PC_71_BM were co-dissolved in chlorobenzene with a weight ratio of 1:1.5 to form the mixed solution with the concentration 20 mg/mL. All organic materials were weighed and dissolved in ambient air conditions.

The devices were fabricated with an architecture of ITO/PEDOT:PSS/PTB7:PC_71_BM/LiF/Al. The indium tin oxide (ITO) glass substrates with a sheet resistance of 10 Ω/Sq were cleaned consecutively in ultrasonic baths containing glass lotion, ethanol and de-ionized water sequentially, and then dried by high pure nitrogen gas. The pre-cleaned ITO substrates were then treated by UV-ozone for 5 min for further cleaning the substrates and improving work function of the ITO substrates. The PEDOT: PSS (purchased from Clevios AI 4083) was spin-coated on the ITO substrates at 3000 rounds per minute (rpm) for 40 s. Then PEDOT: PSS coated ITO substrates were dried in air at 150 °C for 10 min. The substrates were then transferred to a nitrogen-filled glove box (<100 ppm O_2_ and <0.2 ppm H_2_O). On the other hand, in order to fabricate the different devices (without DIO additive) as designed in experiment, 0.5%, 1%, 2.5% and 5% of PS by weight were added into PTB7/PC_71_BM mixed solution respectively only half an hour apart, and then the active layers with different ratios of PS were formed by spin-coating on the PEDOT: PSS with same spin-coating parameters, 1 s for acceleration and 120 s with the rotation speed of 1000 rpm. On the top of the active layer, a 0.7 nm interfacial layer LiF was evaporation deposited under 10^−4^ Pa vacuum conditions. The thickness of LiF was monitored by a quartz crystal microbalance. An aluminum cathode layer about 100 nm was then evaporation deposited on LiF layer under 10^−4^ Pa vacuum conditions in same deposition chamber with changed target. The active area was defined by the vertical overlap of ITO anode and Al cathode which is about 4 mm^2^. The light mask was not used during I-V measurement, and the potential for edge effects may have an effect on the results. For the convenience of discussion, different films and devices were named and prepared to compare their performances:

Film 1: PTB7:PC_71_BM,
Film 2: PTB7:PC_71_BM, 1 wt % PS
Film 3: PTB7:PC_71_BM, 3 v% DIO
Film 4: PTB7:PC_71_BM, 1 wt % PS and 3 v% DIO
Device 1: ITO/PEDOT:PSS/film1/LiF/Al
Device 2: ITO/PEDOT:PSS/film2/LiF/Al
Device 3: ITO/PEDOT:PSS/film3/LiF/Al
Device 4: ITO/PEDOT:PSS/film4/LiF/Al

### 2.2. Photovoltaic Characterization

The absorption spectra of films were measured with a Shimadzu UV-3101 PC spectrometer. The thickness of the active layers is measured by an Ambios Technology XP-2 stylus Profiler. The thicknesses of Film 1, Film 2, Film 3 and Film 4 are 85 nm, 110 nm, 73 nm and 102 nm, respectively. The current–voltage (J-V) characteristics of the OPVs were measured using a Keithley 4200 semiconductor characterization system under a simulated AM 1.5G spectrum with power of 100 mW/cm^2^ generated by ABET Sun 2000 solar simulator. The corresponding J-V curves were recorded from −1 V to 1 V with an interval of 0.01 V. An incident photon to current conversion efficiency (IPCE) spectrum was measured on Zolix Solar Cell Scan 100. The morphology of the films was investigated by atomic force microscopy (AFM) using a multimode Nanoscope IIIa operated in tapping mode. All the samples were measured with a scan size of 5 × 5 µm^2^. The hole mobility and electron mobility of PTB7: PC_71_BM blend films were measured by space charge limited current (SCLC) method. All the tests were in ambient air conditions.

## 3. Results and Discussions

**Figure 1:**
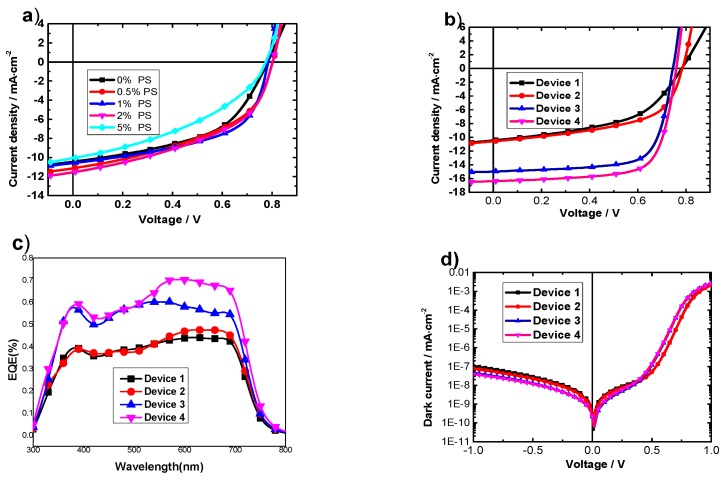
(**a**) The J-V characteristic curves of solar cells with different doping ratios of PS under AM 1.5 light power of 100 mW/cm^2^; (**b**) The J-V characteristic curves of solar cells without or with 3 v% DIO and/or 1 wt % PS; (**c**) the external quantum efficiency (EQE) of the devices in the system of PTB7:PC71BM without or with 3 v% DIO and/or 1 wt % PS; (**d**) The J-V characteristics cast from solar cells without or with 3 v% DIO and/or 1 wt % PS in darkness.

The J-V characteristic curves of the OPVs with different PS ratios are shown in Figure 1a. The PV performances of the OPVs are summarized according to the J-V curves and listed in Table 1. Among all the different ratios, it can be found that the device with 1 wt % of PS demonstrates the highest median PCE of 4.56% along with a short-circuit current (*J_sc_*) of 10.60 mA/cm^2^, an open-circuit voltage (*V_oc_*) of 0.79 V, and a fill factor (FF) of 54.50%. The data in Table 1 shows that the PCE improvement is mainly attributed to the enhancement in *J_sc_* and FF. To further investigate the mechanism responsible for the enhanced performance of the OPVs with the PS additions, the optimized volume ratio of 1% was used.

It is reported that DIO can improve the morphology of the active layer and enhance the performance of organic solar cells [24]. Consequently, organic solar cells based on PTB7:PC_71_BM with two additives DIO and PS were prepared to improve photovoltaic properties. The concentration of DIO is 3 wt % according the reference [18], and that of PS is 1 wt % according to the above results. The J-V curves of the OPVs with different additives under illumination of simulated AM1.5G (100 mW/cm^2^) are shown in Figure 1b and summarized in Table 2. Device 1 demonstrates a PCE of 4.11% with a *J_sc_* of 10.47 mA/cm^2^, a *V_oc_* of 0.79 V, and a FF of 49.65%.

As shown in Table 2, with the addition of 1 wt % PS (weight fraction of the BHJ components) in Device 2, *J_sc_* increases to 10.60 mA/cm^2^ and FF increases to 54.50%, which results in a PCE of 4.56%. If both 3.0 v% DIO and 1 wt % PS are added to the solution prior to spin casting, the PCE of Device 4 is further increased to 8.92 along with a *V_oc_* of 0.76 V, a *J_sc_* of 16.37 mA/cm^2^, and a FF of 71.68%. The improved *J_sc_* value is confirmed by measuring EQE (Figure 1c). The maximum EQE value of Device 1 is 43.72% and it is increased to 63.37% for Device 4. The single logarithmic dark current curves show that Device 4, Device 3 and Device 2 have smaller leakage current compared with Device 1, as shown in Figure 1d. It is well-known that the leakage current is determined by the shunt resistance (R_sh_) [25]. The larger R_sh_ indicates a lower charge carrier recombination in the active layer. This indicates that DIO and/or PS can effectively restrain the leakage current under reverse bias, which may provide effective charge carrier transport in the blend layers and result in an increase of *J_sc_* compared to that of Device 1. The smaller R_s_ indicates a lower resistance of the semiconductor bulk resistance and a better metal/semiconductor interface connection induced by using additives [25].

**Figure 2:**
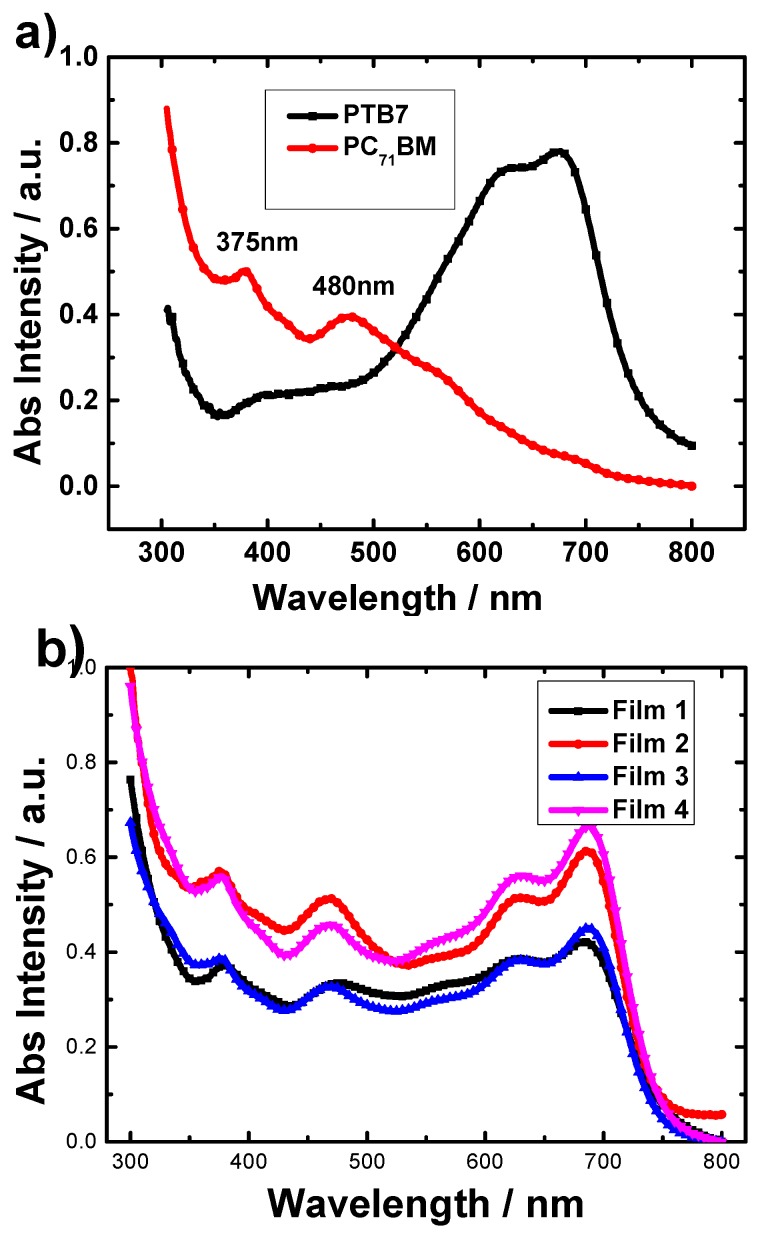
(**a**) The absorption spectra of neat PTB7 and PC_71_BM films; (**b**) absorption spectra of PTB7/PC_71_BM films cast from solvents with or without DIO and/or PS additives. The data was normalized by the film thickness.

Under the same spin-coating condition, the thicknesses of films 1 and 3 are almost the same. When doped with PS, the thicknesses of films 2 and 4 increases and are almost the same. This shows that doping PS encourages an increase the thickness of the active layer without decreasing other electronic properties; for example, Rsh and Rs of corresponding devices doped with PS are improved as shown in Table 2. In order to understand the effect of high-molecular-weight insulating polymers-PS in the mixture of PTB7 and PC_71_BM on the optics properties and the surface morphology, further characterization has been carried out. The absorption spectra of the neat PC_71_BM and PTB7 films are shown in Figure 2a. PC_71_BM has two apparent absorption peaks at 375 nm and 480 nm. The absorption spectra of PTB7 show an apparent complementary absorption in the range from 550 nm to 750 nm. Two broad absorption peaks at around 624 and 682 nm are attributed to the characteristic π-π* transition of the PTB7 polymer [26,27]. In comparison to the absorption spectra of the films prepared with and without additives, there is no obvious peak shift observed in the film prepared with PS and in the film prepared with pristine CB, as shown in Figure 2b.

It can be found there is no significant difference between Film 1 and Film 3, while there is an increase of obvious relative absorption intensity in the region of 300–800 nm for Film 2 and Film 4. This result indicates that under the same conditions, films with PS additives can harvest solar photons more effectively than the films prepared from CB and CB: DIO. The more light absorbed, the higher photocurrent generated [28]. This is the reason why the *J*_sc_ increases after the addition of high-molecular-weight insulating polymer PS.

**Figure 3:**
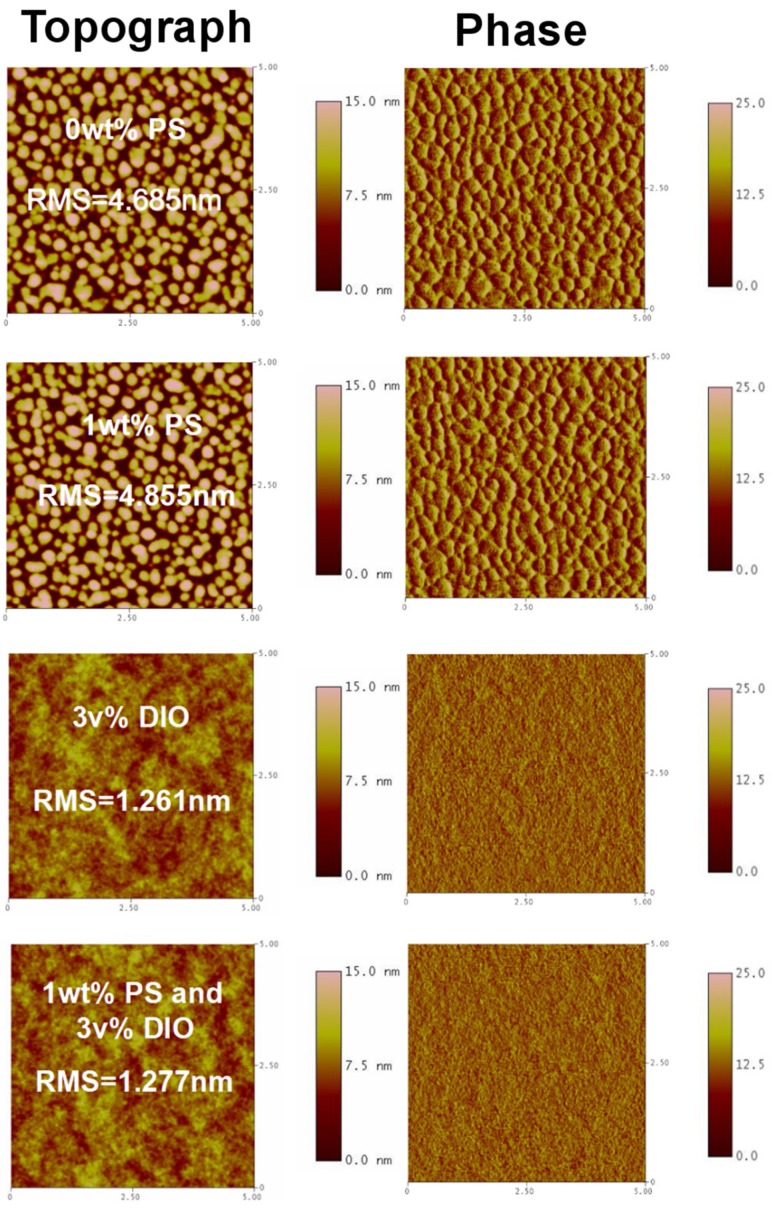
The AFM surface topography and phase images of the PTB7:PC_71_BM films with/without 3 v% DIO and/or 1 wt % PS.

In order to investigate the effects of PS and DIO on the morphology of the blend films, the surface topography and phase images of the blend films have been studied by atomic force microscopy (AFM) in taping mode (5 μm × 5 μm), as shown in Figure 3. The roughness of Film 1, Film 2, Film 3 and Film 4 are 4.685, 4.855, 1.261 and 1.277 nm, respectively. Obviously, the roughness of the blend films decrease after the addition of DIO, and the phase separation are more finely compared with Film 1 and Film 2, as shown in the phase images. The addition of DIO to the casting solvent results in smaller domains and a more finely interpenetrating BHJ morphology, relative to blend films cast without DIO as shown in the phase diagrams. In particular, Film 4 does not reveal significant increases in roughness at the nanoscale compared to Film 3. Also, the film roughnesses of Film 1 and Film 2 are very similar. All this indicates that incorporating the insulating PS within the photovoltaic layer without negative drawbacks in phase separation.

**Figure 4:**
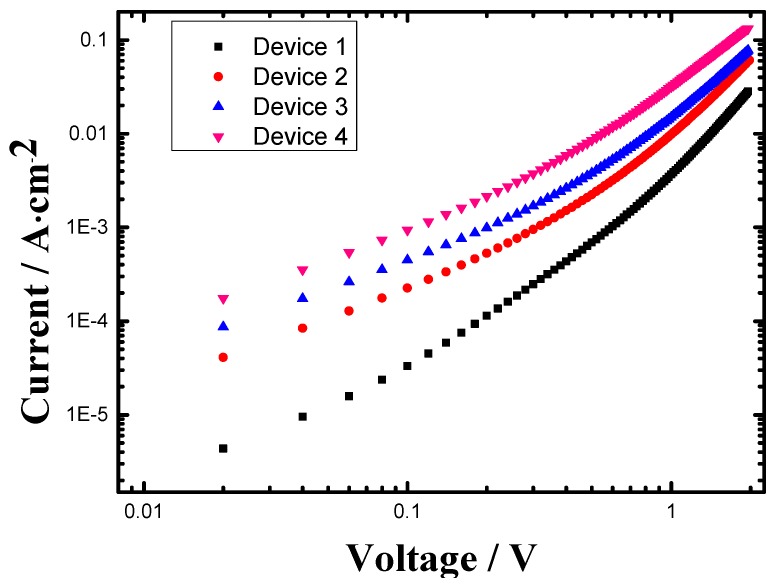
J-V characteristic curves of hole-only devices with/without 3 v% DIO and/or 1 v% PS.

In order to further investigate the effects of additive on the charge carrier transport, the hole-only devices have been fabricated based on pristine PTB7 films and blend PTB7:PC_71_BM films with/without additives, respectively. High-work-function material gold (Au) is used as the cathode to block the back injection of electrons. The dark J-V curves of the hole-only devices with the configuration of ITO/PEDOT:PSS/PTB7/Au and ITO/PEDOT:PSS/PTB7:PC_71_BM/Au are measured and shown in Figure 4. The hole transport through the polymer film is limited due to the accumulation of space charge when a sufficient voltage is applied to this hole-only device. The space charge limited current(SCLC) is described by the equation [29,30]:

(1) $$J=\frac{9}{8}\varepsilon \mu \frac{V^{2}}{d^{3}}=\frac{9}{8}\varepsilon_{0}\varepsilon_{r}\mu \frac{V^{2}}{d^{3}}$$

where *ε_0_* is the permittivity of free space, ε_r_ is the dielectric constant of the blend material, *μ* is the hole mobility, *V* is the voltage drop across the device and *d* is the active layer thickness. The parameter *ε_r_* is assumed to be 3, which is a typical value for conjugated polymers. The hole will be collected by the ITO electrode, which is very similar to hole transport process in the OPVs. The J-V characteristics of neat PTB7 and PTB7:PC_71_BM with/without additives of 3 v% DIO and 1 wt % PS fully agree with the SCLC model. According to the J-V curves, the hole current density of the hole-only devices with 1 wt % PS is larger than that of the hole-only devices without any additive, the hole current density of the hole-only devices with 3 v% DIO is larger than that of the hole-only devices with 1 wt % PS and the hole current density of the hole-only devices with additives of both 3 v% DIO and 1 wt % PS is larger than that of the hole-only devices with only 3 v% DIO. This means that hole carrier transport in the hole-only devices with 3 v% DIO and/or 1 wt % PS has been improved compared with that of the hole-only devices without any additive. The result indicates that even though the thickness of the film with PS is greater than that without PS in the same fabricate condition, the hole mobility of the device with PS is better than that without PS, which shows PS can be good for hole carrier transport. Moreover it further demonstrates that the improved hole carrier transport could be one of the reason for the increased *J_sc_* of OPVs [30,31].

## 4. Conclusions

A series of OPVs with PTB7:PC_71_BM as the active layer are fabricated to investigate the additive’s effects on the performance of the OPVs. DIO and PS are used as the additives. The experimental results of photovoltaic performance reveal an enhancement of *J_sc_* from 10.47 to 16.37 mA/cm^2^ and FF from 49.65% to 71.68% by adding DIO and PS. As a result, the PCEs of the OPVs are improved from 4.11% to 8.92%, with 117% improvement compared with the OPVs based on PTB7:PC_71_BM without additives. The positive effect of DIO and PS additives on the performance of the OPVs should be attributed to the increased absorption and charge carrier transport and collection.

## Figures and Tables

**Table 1:** The PV performance of ITO/PEDOT: PSS/PTB7:PC71BM/LiF/Al photovoltaic devices with different doping ratios of PS.

Doping Ratio	*V_oc_* (V)	*J_sc_* (mA/cm^2^)	FF (%)	PCE (%)
0 wt %	0.79 ± 0.01	10.47 ± 0.09	49.65 ± 0.05	4.11 ± 0.02
0.5 wt %	0.79 ± 0.01	11.15 ± 0.08	46.61 ± 0.06	4.16 ± 0.02
1 wt %	0.79 ± 0.01	10.60 ± 0.08	54.50 ± 0.04	4.56 ± 0.02
2 wt %	0.80 ± 0.01	11.55 ± 0.09	46.68 ± 0.05	4.31 ± 0.02
5 wt %	0.80 ± 0.01	10.07 ± 0.13	38.74 ± 0.08	3.12 ± 0.03

**Table 2:** The summary of photovoltaic parameters of PTB7:PC71BM system solar cells without or with 3 v% DIO and/or 1 wt % PS.

	*V_oc_* (V)	*J_sc_* (mA/cm^2^)	FF (%)	PCE (%)	Rsh (Ωcm^2^)	Rs (Ωcm^2^)
Device 1	0.79 ± 0.01	10.47 ± 0.013	49.65 ± 0.02	4.11 ± 0.03	302.8	18.2
Device 2	0.79 ± 0.01	10.60 ± 0.012	54.50 ± 0.09	4.56 ± 0.02	311.5	8.56
Device 3	0.75 ± 0.01	14.23 ± 0.09	71.31 ± 0.05	7.61 ± 0.02	757.6	5.44
Device 4	0.76 ± 0.01	16.37 ± 0.08	71.68 ± 0.04	8.92 ± 0.02	915.8	4.24
